# Synthesis and Characterization of Starch-Based Acid- and Alkali-Resistant Hydrogels Optimized by Box–Behnken Response Surface Methodology

**DOI:** 10.3390/gels8090585

**Published:** 2022-09-15

**Authors:** Xiaoxue Han, Lijie Huang, Qi Mo, Zhehao Wei, Yanan Wang, Yishan Li, Chongxing Huang, Qingshan Duan, Yingnan Wei

**Affiliations:** 1 College of Light Industry and Food Engineering, Guangxi University, Nanning 530004, China; 2 Guangxi Key Laboratory of Clean Pulp & Papermaking and Pollution Control, Nanning 530004, China; 3 Guangxi Bossco Environmental Protection Technology Co., Ltd., Nanning 530007, China

**Keywords:** hydrogels, mechanical properties, response surfaces, acid and alkali resistance

## Abstract

Applying gel-type solid chlorine dioxide for the sustained release of chlorine dioxide has several shortcomings, such as no resistance to acid and alkali corrosion and poor mechanical properties. However, introducing quaternary ammonium, carboxyl, and amino groups into the hydrogel system can enhance its acid and alkali resistance. In this study, the effects of concentration of dry heat-modified starch, quaternized carboxymethyl cellulose, and chitin on the swelling behavior and mechanical properties of starch-based acid- and alkali-resistant hydrogels are investigated. The feasibility of the actual and predicted values of the tentative results is verified based on the response surface design to determine the optimal concentration ratio of acid- and alkali-resistant hydrogels. The results reveal that optimized process parameters are reliable. The maximum swelling ratio and compressive stress of the hydrogel are 5358.00% and 44.45 kPa, respectively, and its swelling behavior conforms to the pseudo second-order kinetic model. Thus, the present study can provide a new method of developing efficient starch-based chlorine dioxide hydrogels for the sustained release of chlorine dioxide.

## 1. Introduction

Hydrogel is a vast three-dimensional (3D) hydrophilic polymer network capable of swelling large amounts of water or other fluids [1,2]. Depending on the material source, hydrogels can be classified into synthetic, natural, and “hybrid” polymers [3,4]. Hybrid hydrogels are based on natural and synthetic polymer/monomer combinations, which can generate more functional groups and strengthen the mechanical properties of natural polymers [5,6]. These hydrogels have several applications such as in chlorine dioxide sustained release and wastewater adsorption [7,8]. In the slow release of chlorine dioxide, solid chlorine dioxide products with gel organics as carriers can stably release chlorine dioxide over an extended period [9]. Although utilizing biomass materials such as cellulose [10] in chlorine dioxide slow release is currently of great interest, several problems are associated with solid chlorine dioxide products such as no acid and alkali resistance and that they are easily collapsible and disintegrable in acids and alkalis. Hence, exploring long-acting and prolonged-release solid chlorine dioxide hydrogels with acid and alkali resistance and excellent mechanical properties is necessary.

The number of studies available on the application of acid- and alkali-resistant hydrogels in the sustained release of chlorine dioxide is relatively few. Nevertheless, enhancing the porosity in the polymer carrier material and increasing the number of hydroxyl, carboxyl, and quaternary ammonium groups can strengthen the resistance of hydrogels to acid and alkali solutions. Enhancing the internal resistance of the carrier material to slow down the binding rate of sodium chlorite and active acid can effectively improve acid and alkali resistance. As a biodegradable biomass resource, cassava starch can save chlorine dioxide sustained release application costs [11,12,13]. This can be achieved by regulating the network structure, skeleton structure, and functional groups of the hydrogel, combined with the chemical structure of the hydrogel and the molecular level of the cross-linked network to design the acid- and alkali-resistant solid chlorine dioxide hydrogel, thus improving the defects of the existing materials.

In this study, the effects of different component concentrations (dry heat-modified starch, quaternized carboxymethyl cellulose, chitin, polyvinyl alcohol (PVA), sodium bicarbonate, and epichlorohydrin) on the swelling properties and compression performance of the hydrogel were investigated. The raw material concentration ratio that influences the performance of the hydrogel was optimized by the response surface method. Because the real value was close to that of the result predicted by the response surface, the process parameters obtained by the experimental optimization were reliable. The chemical structure, appearance, and swelling behavior of hydrogels were examined using Fourier-transform infrared (FTIR), scanning electron microscopy (SEM), and swelling kinetics. Thus, the present study can provide a new method of developing efficient starch-based chlorine dioxide hydrogels for the sustained release of chlorine dioxide that can be used for environmental disinfection.

## 2. Results and Discussion

### 2.1. Formation Mechanism of Hydrogels

**Figure 1:**
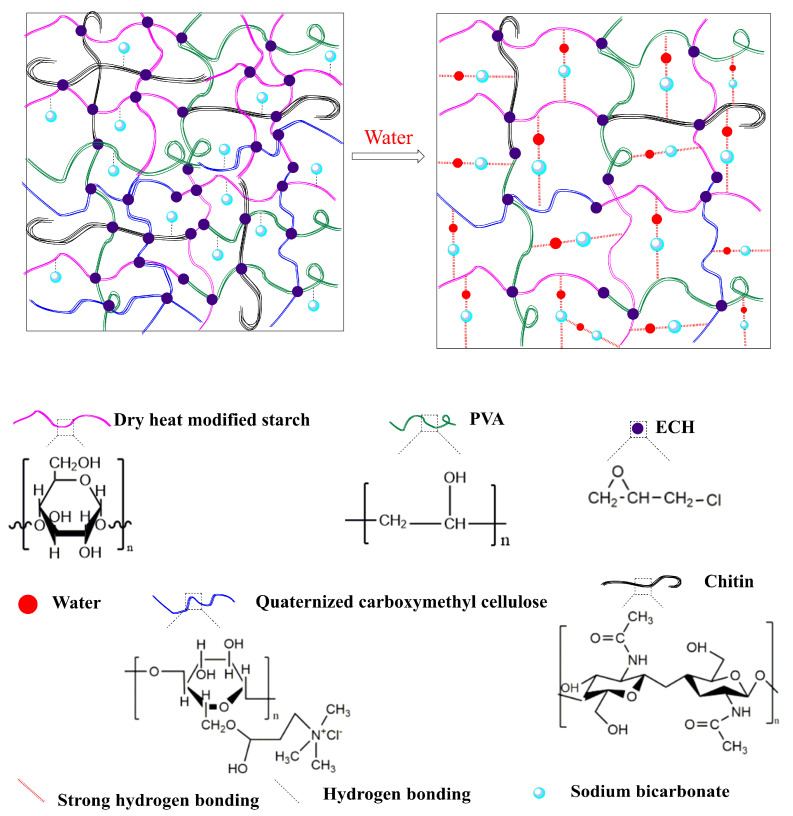
A schematic diagram of the structure and mechanism of acid- and alkali-resistant hydrogels.

The synthesis mechanism of the starch-based acid- and alkali-resistant hydrogels involves chemical cross-linking reactions. The characteristics of chemical crosslinking enable the formation of a rigid and firm hydrogel. Theoretically, the crosslinked networks tying together long linear polymer molecules to give an infinite network structure from bigger molecules creates congestion in the solution system [14]. Dry heat modifies starch, chitin, quaternized carboxymethyl cellulose, and polyvinyl alcohol through sodium hydroxide alkalization and epichlorohydrin cross-linking reactions, with the introduction of sodium bicarbonate in the hydrogel system, and finally the formation of a hydrogel. The formation mechanism is mainly that the cross-linking reaction occurred with epichlorohydrin in the created alkaline environment, and Epichlorohydrin as a cross-linking agent broke the carbon–chlorine bond at one end, forming a carbon-positive ion to form ether with the hydroxyl group of the dry heat-modified starch. The epoxide at the other end opened the ring and formed ether with the hydroxyl group of the quaternized carboxymethyl cellulose, then the epichlorohydrin broke the carbon–chlorine bond at one end, forming a carbon-positive ion to form ether with the hydroxyl group of the chitin molecule; the other end of the epoxide opens the ring to form ether with the hydroxyl group of polyvinyl alcohol molecule, so as to obtain the cross-linked product macromolecule which is the target product hydrogel. As shown in Figure 1.

### 2.2. Effect of Different Component Concentrations on the Swelling Ratio of Hydrogels

**Figure 2:**
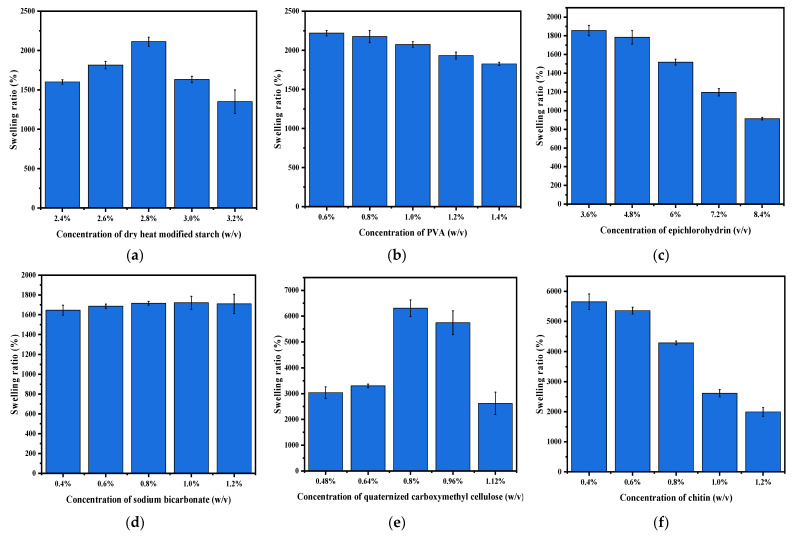
Swelling ratios of hydrogels with different component concentrations in distilled water: (**a**) dry heat-modified starch, (**b**) polyvinyl alcohol, (**c**) epichlorohydrin, (**d**) sodium bicarbonate, (**e**) quaternized carboxymethyl cellulose, and (**f**) chitin.

As shown in Figure 2a,d,e, the swelling ratio exhibits an increasing and then decreasing tendency with an increase in the concentrations of dry heat-modified starch, sodium bicarbonate, and quaternized carboxymethyl cellulose. This tendency was observed because of the large number of reactive groups on the surface of the dry heat-modified starch that produced a denser hydrogel network structure [11,15] and restricted the entry of water molecules. The addition of quaternized carboxymethyl cellulose enriched the hydrogel system with quaternary ammonium cations (NH_4_^+^) and carboxylic acid anions (COO^−^), resulting in an electrostatic attraction that was conducive to establishing the gel network. Subsequently, the network interaction became stronger as the concentration increased, thus decreasing the swelling ratio. The formation of bubbles is retained in the polymer network when the amount of sodium bicarbonate in the hydrogels is increased [16]. This resulted in more porous structures [17], thus increasing the swelling ratio of the hydrogel. However, when the concentration of sodium bicarbonate is higher, excess pores are formed, and the pore walls become thinner, thus decreasing the swelling ratio [18]. In contrast, a decreasing trend is observed in Figure 2b,c,f with an increase in PVA, epichlorohydrin, and chitin concentrations. This is because the hydrogen bonding with the dry heat-modified starch becomes stronger with the excess PVA, and the physical cross-linking of the network increases. Thus, the hydrogel structure becomes dense, and the internal structure is unable to accommodate the large amount of water. In the case of epichlorohydrin, more cross-linked networks were formed in the hydrogel solution system with an increase in concentration, resulting in a hard and firm physical structure of hydrogel [19,20]. The cross-linked density of the hydrogel also increased with an increase in the chitin concentration [21], thus decreasing the gel-swelling ratio with increasing gel cross-linking.

### 2.3. Mechanical Test Analysis

**Figure 3:**
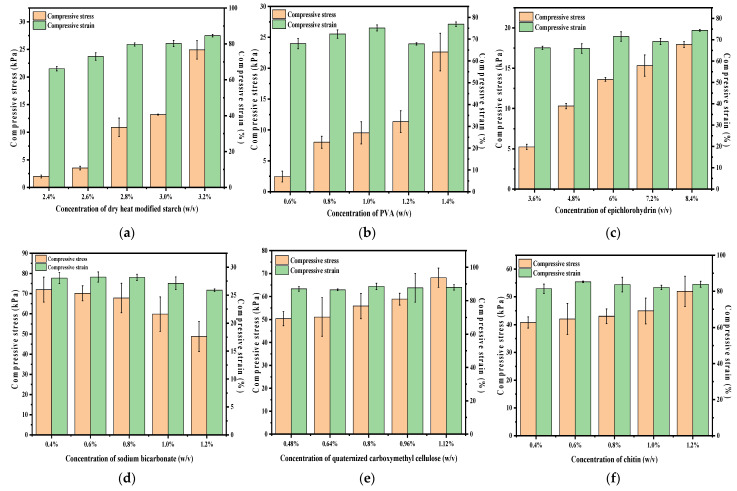
Compression tests of hydrogels with different component concentrations: (**a**) dry heat-modified starch, (**b**) polyvinyl alcohol, (**c**) epichlorohydrin, (**d**) sodium bicarbonate, (**e**) quaternized carboxymethyl cellulose, and (**f**) chitin.

As illustrated in Figure 3a–c,e,f, the maximum detectable compressive stress of the hydrogels significantly increased with increasing dry heat-modified starch, PVA, epichlorohydrin, quaternized carboxymethyl cellulose, and chitin. Initially, the dry heat-modified starch significantly improved the compressive resistance of the hydrogels with many hydroxyl groups on their surface. These hydroxyl groups formed hydrogen bonds with PVA chains to form a cross-linked network with chain entanglement [22]. The number of entangled cross-linking sites and hydrogen bonds was larger when the molecular weight of PVA was higher due to the formation of inter- and intra-chain hydrogen bonds; the entanglement effect enhanced the stable cross-linked hydrogel network [23]. Subsequently, the cross-linking reaction rate was considerably increased with an increase in the concentration of epichlorohydrin. This resulted in the formation of more interlocked cross-linked networks between the polymers in the solution system, which limited the molecular mobility. The concentration of quaternary ammonium cations in the hydrogel increased with an increase in the concentration of quaternized carboxymethyl cellulose. The cations combined with the carboxylic acid anions, resulted in an electrostatic interaction. This led to an increase in the degree of cross-linking and rigidity within the hydrogel, which formed a tighter cross-linking network [24], covalent bonds with the PVA chains as soft segments, and a network that reduced the mobility of the polymer chains [25]. Chitin has a large number of hydrophilic groups, such as hydroxyl and amino groups, attracting the water molecules into the network lattice. Thus, the number of cross-linking points was more, and the hydrogel network structure was denser when the chitin concentration was higher. Moreover, the compressive stress increased with increasing cross-linking density. In the case of sodium bicarbonate (Figure 3d), the compressive stress and compressive strain of the hydrogel exhibited a decreasing trend with increasing concentration. This is because with an increase in the sodium bicarbonate concentration, the thermal decomposition of HCO_3_^−^ releases carbon dioxide, which forms pores within the hydrogel and increases the gel permeability. Thus, more porous structures are formed inside and on the surface of the hydrogel, decreasing the mechanical properties of the hydrogel.

### 2.4. Box—Behnken Design Optimization of Swelling and Compression Performance

The response surface design of the central combination test and the results are illustrated in Table 1.

#### 2.4.1. Analysis of Variance (ANOVA) with Swelling Properties as the Response Value

The values of reaction parameters NaOH (6%), epichlorohydrin (6%), PVA (1%), and sodium bicarbonate (0.6%) were fixed to achieve the maximum percentage of swelling. The concentration of dry heat-modified starch, quaternized carboxymethyl cellulose, and chitin were important parameters that affected the swelling ratio. Hence, the interaction of these parameters was analyzed using a Box—Behnken design with the swelling properties (Y_1_) as the response value. The following was the coded regression equation:

Y_1_ = +5142.40 + 242.38A + 352.12B − 146.50C − 397.00AB − 354.25AC − 191.25BC − 1011.20A^2^ − 1246.20B^2^ − 912.45C^2^ (1)

where A, B, and C indicate dry heat-modified starch, quaternized carboxymethyl cellulose, and chitin, respectively. The regression equation for the actual true value was as follows:

Y_1_ = −282,671 + 632,074A + 47,3647B + 224,879C − 198,500AB − 141,700AC − 956,25BC − 404,480A^2^ − 778,875B^2^ − 364,980C^2^ (2)

The significance and accuracy of the regression model were evaluated based on the regression equations of the above coded and true values, as shown in Table 2. The probability of the underfitting test of the equation was 0.1022, indicating that the model misfit was insignificant [26]. Conversely, the linear relationship between the dependent variable and all independent variables was significant when the regression equation was used to describe the relationship between the factors and the response values. Because the regression equation could be used to determine the best preparation process, the model is valid for this study.

**Figure 4:**
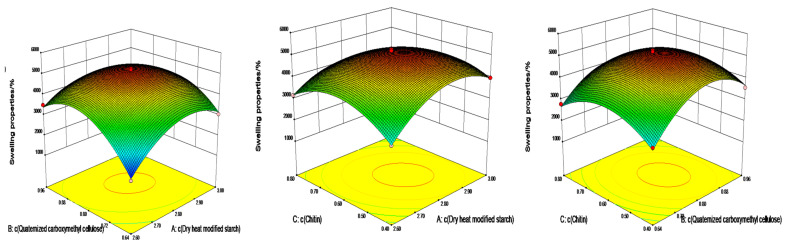

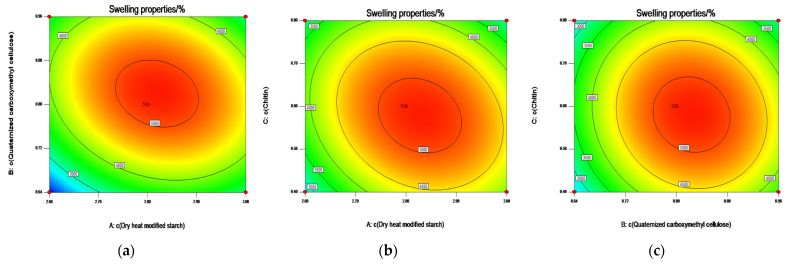
Contour and 3D response plots showing (**a**) the effects of concentration of dry heat-modified starch (A), concentration of quaternized carboxymethyl cellulose (B), and their interaction on the swelling properties; (**b**) concentration of dry heat-modified starch (A), concentration of chitin (C), and their interaction on the swelling properties; (**c**) concentration of quaternized carboxymethyl cellulose (B), concentration of chitin (C), and their interaction on the swelling properties.

Combine the F values of AB, AC, and BC in Table 2, the 3D response surface and two-dimensional (2D) contour plots were used to explain the interactions of dry heat-modified starch (A), quaternized carboxymethyl cellulose (B), and chitin (C) concentrations on the swelling properties. As shown in Figure 4, the contour plot in Figure 4a is tighter, indicating the significant effect of the interaction between chitin and quaternized carboxymethyl cellulose on the swelling properties. The interaction between chitin and the dry heat-modified starch exhibited the second-highest effect on the swelling properties. Compared to the interactions mentioned above, the interaction between the dry heat-modified starch and quaternized carboxymethyl cellulose had less effect on the swelling properties.

#### 2.4.2. ANOVA with Compression Performance as Response Value

To achieve the maximum compressive stress, the values of fixed reaction parameters considered were the same as that in Section 2.4.1. The dry heat-modified starch, quaternized carboxymethyl cellulose, and chitin concentrations were simultaneously important parameters that affected the compressive stress, with the compressive properties (Y_2_) as the response value. The regression equation after coding by the Box–Behnken design analysis was as follows:

Y_2_ = +44.66 − 3.84A − 2.27B + 1.51C + 3.30AB − 3.58AC + 1.91BC − 9.72A^2^ − 6.85B^2^ − 10.11C^2^ (3)

where A, B, and C indicate dry heat-modified starch, quaternized carboxymethyl cellulose, and chitin, respectively. The regression equation for the actual true value was as follows:

Y_2_ = −1952.77 + 5250.20A + 358.70B + 2056.73C + 1650.00AB − 1433.90AC + 953.00BC − 3887.01A^2^ − 4283.61B^2^ − 4044.61C^2^ (4)

The above model was tested for the lack of fit. As shown in Table 3, the probability of insufficient fitting of the equation was 0.6621. This result indicates that the lack of fit of the model is insignificant and that the model fits well. The above regression equation describes the relationship between each factor and the response value. When a relationship is present, the linear relationship between the dependent variable and all independent variables is significant; hence, the equation is significant. In Table 3, the P values of items A, B, C, AC, AB, BC, A^2^, B^2^, and C^2^ are less than 0.05, which is significant [27]. In addition, the *p* values of items A, B, AC, AB, A^2^, B^2^, and C^2^ are less than 0.01, which is highly significant. Because each factor has a significant effect on the compressibility of the hydrogel, the regression equation can be used to determine the optimal extraction process conditions. Thus, the experimental results can be analyzed and predicted.

**Figure 5:**
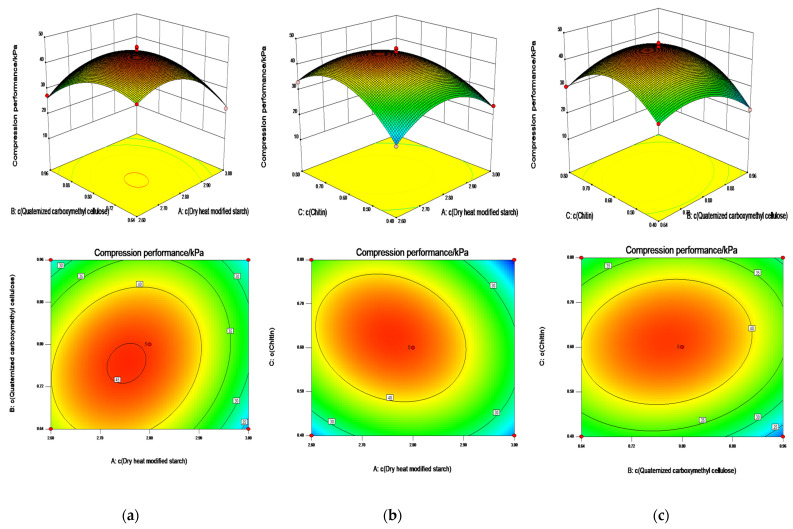
Contour and 3D response plots showing the effects of (**a**) concentration of dry heat-modified starch (A), concentration of quaternized carboxymethyl cellulose (B), and their interaction on the compression performance; (**b**) concentration of dry heat-modified starch (A), concentration of chitin (C), and their interaction on the compression performance; (**c**) concentration of quaternized carboxymethyl cellulose (B), concentration of chitin (C), and their interaction on the compression performance.

As shown in Figure 5, the contour plot in Figure 5a is tighter compared to that in Figure 5b,c, indicating that the interaction effect of dry heat-modified starch and quaternized carboxymethyl cellulose has a greater influence on the compression performance. The compression performance of the hydrogel exhibited a gradual increase with an increase in the concentrations of dry heat-modified starch, quaternized carboxymethyl cellulose, and chitin. The regression equation is in good agreement with the experimental results, indicating that the model is accurate and meets the optimization requirements.

Three repetitive tests were conducted under the process conditions of concentration of dry heat-modified starch is 2.8%, the concentration of quaternized carboxymethyl cellulose is 0.8%, and the concentration of chitin is 0.6%, to prove the feasibility of response surface optimization further. The predicted value of the swelling performance was 5085.25%, while the actual measured swelling performance was 5358.00%, with an error value of 4.9%. Additionally, the predicted value of the compression performance was 41.85 kPa, whereas the actual measured real value was 44.45 kPa, with an error value of 5.8%. The real value was consistent with the predicted value of the model, which confirmed that the process parameters obtained from the experimental optimization were reliable. Thus, the accuracy and validity of the process parameter model were illustrated.

### 2.5. Swelling Kinetics

The swelling kinetic models were screened to investigate the swelling properties of the hydrogels further. The pseudo first- and secondary-order kinetic models were employed to investigate the swelling properties.

The pseudo first-order kinetic model for the absorption rate in hydrogel swelling is illustrated in Equation (5) [28].

(5) $$\ln(Q_{e}-Q_{t})=\ln Q_{e}-k_{1}t$$

where *Q_e_* and *Q_t_* are the equilibrium water uptake at equilibrium and moment *t* (g/g), and *k*_1_ is the kinetic constant (g/(g·min)).

The pseudo second-order kinetics of the absorption rate in hydrogel swelling is modeled according to Equation (6) [29].

(6) $$\frac{t}{Q_{t}}=\frac{1}{kQ_{e}^{2}}+\frac{t}{Q_{e}}$$

where *Q_t_* and *Q_e_* are the equilibrium water uptake at equilibrium and moment *t* (g/g), and *k* is the dissolution rate constant; *K_c_* = *k* × *Q_e_*^2^, which indicates the initial dissolution rate constant.

**Figure 6:**
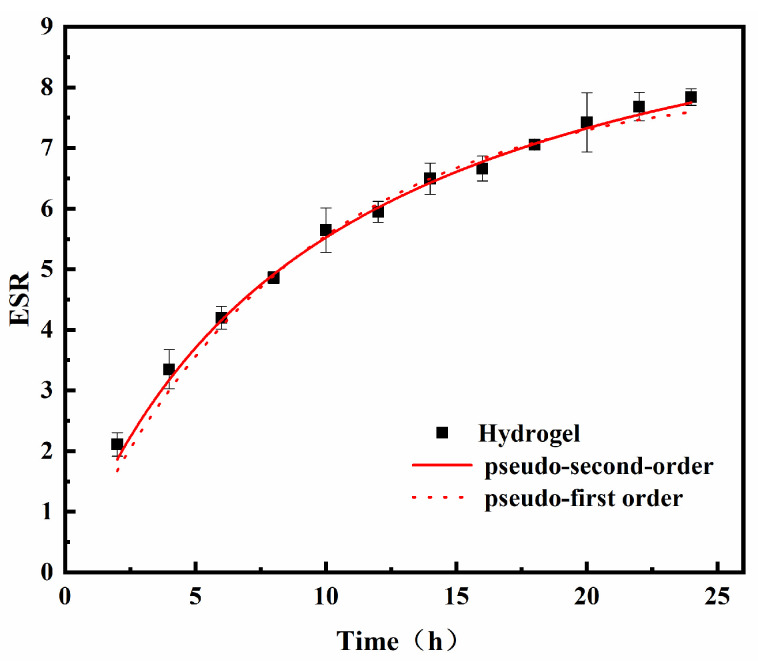
Swelling kinetic curve of the hydrogel.

As illustrated in Figure 6 and Table 4, the swelling curves of the hydrogels were fitted in the pseudo first- and secondary-order kinetic models, respectively, and the pseudo second-order kinetic model was found to be a better fit (R^2^ = 0.997). The pseudosecond-order model can describe the swelling of hydrogels in distilled water, this is because the swelling of hydrogels in distilled water causes their carboxyl groups to be ionized and electrostatic repulsions between molecular chains to dominate the system, leading to more swelling of the network. In addition, the osmotic pressure increases with the rate of absorption in the initial stage, facilitating faster swelling of the hydrogel network, and the rate of swelling between the hydrogel and distilled water is controlled mainly by chemical reactions [30,31,32].

### 2.6. Scanning Electron Microscopy

**Figure 7:**
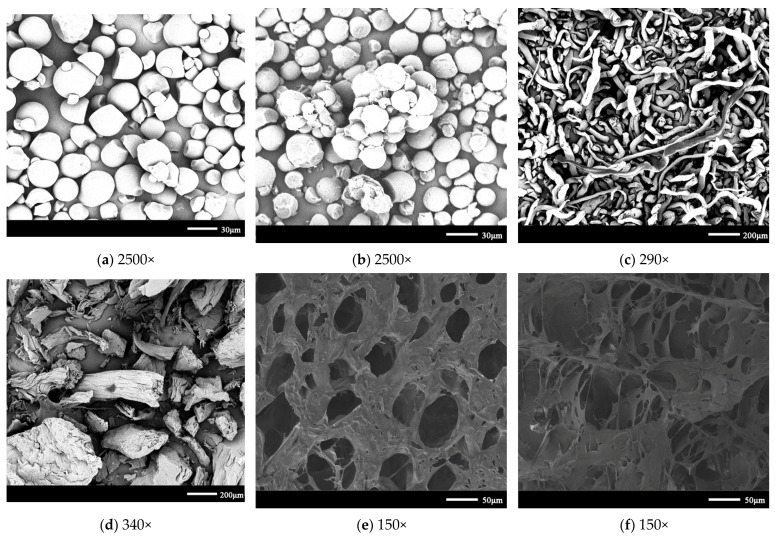
Scanning electron microscopy: (**a**) cassava starch, (**b**) cassava starch after dry thermal modification, (**c**) carboxymethyl cellulose, (**d**) quaternized carboxymethyl cellulose, (**e**) surface of hydrogel, and (**f**) cross-section of hydrogel.

As shown in Figure 7a,b, the cassava starch granules were spherical, oval, or oval truncated, with an average particle size of 15 μm and a range of 5–20 μm [33]. The shape of the cassava starch granules modified by the dry heat showed no change, with depression, adhesion, and agglomeration on the surface. This phenomenon occurred because the xanthan gum interacted with the straight chain starch outside the starch granules due to heating and pre-modification. The SEM before and after modification showed that the modification slightly affected the size of the starch granules [34]. As shown in Figure 7c,d, the carboxymethyl cellulose demonstrated ribbons and fibers with a curled surface [35]. The surface of the quaternized carboxymethyl cellulose was rough and uneven, and the original cellulose shape was not maintained due to the grinding and other operations during the treatment. From the surface and cross-sectional morphology of the hydrogel in Figure 7e,f, the surface was observed to be relatively rough [36], and many pores were observed on the pore wall. These pores interconnected between the internal pores to form larger interconnected pores, which was consistent with the analysis described in Section 2.3. The hydrogel produced a dendritic structure after the addition of sodium bicarbonate, which can pass through the tiny CO_2_ bubbles generated during the hydrogel preparation, and change the internal structure [37].

### 2.7. FTIR Analysis

**Figure 8:**
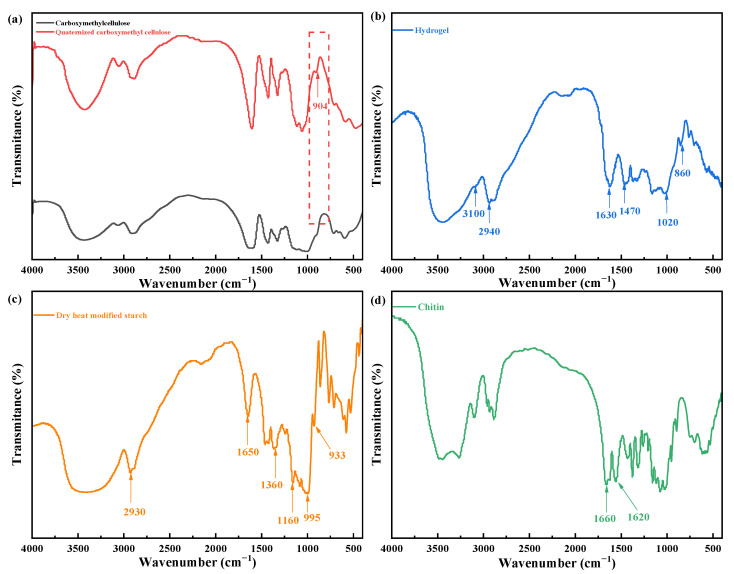
Fourier-transform infrared spectra of the hydrogel and raw materials. (**a**) carboxymethylcellulose (black line) and quaternized carboxymethylcellulose (red line), (**b**) Hydrogel, (**c**) dry heat modified starch and (**d**) Chitin.

As shown in Figure 8, the dry heat-modified starch exhibited a vibrational absorption peak at 2930 cm^−1^ caused by the stretching of the C–H bond; the peak at 1650 cm^−1^ is attributed to the stretching of the C=O bond, while the peak at 1360 cm^−1^ is ascribed to the bending vibrational characteristic of C–H. The peaks at 1180–953 cm^−1^ were typical absorption peaks for starch [38]. Compared to carboxymethyl cellulose, a new peak at 904 cm^−1^ was observed for the quaternized carboxymethyl cellulose, indicating the successful introduction of the quaternary ammonium group into carboxymethyl cellulose by grafting or cross-linking [39]. The amide I bands were observed at 1660 cm^−1^ and 1620 cm^−1^ for chitin, which is ascribed to the C=O secondary amide stretching [40]. The characteristic peaks of the dry heat-modified starch were observed at 2940, 1630, and 1020 cm^−1^ for the hydrogel. A new peak of quaternized carboxymethyl cellulose was demonstrated at 860 cm^−1^, while a peak of the N–H secondary amide asymmetry of chitin was observed at 3100 cm^−1^. Additionally, the corresponding peak of the N–H bending vibration and C–N stretching vibration was observed at 1470 cm^−1^, indicating the interaction between the individual raw materials in the hydrogel.

## 3. Conclusions

In this study, the effects of concentration of dry heat-modified starch, quaternized carboxymethyl cellulose, and chitin on the swelling properties and compression performance of the hydrogel were investigated. The raw material ratio of the acid- and alkali-resistant hydrogel was optimized using the response surface method, providing a reference for studying the effects of the swelling behavior of this hydrogel in acid and alkali solutions. Furthermore, it presents a theoretical basis for application in chlorine dioxide slow release, such as environmental disinfection. The best raw material ratio of the hydrogel is 2.8% of dry heat-modified starch, 0.8% of quaternized carboxymethyl cellulose, 1.0% of polyvinyl alcohol, 6.0% of epichlorohydrin is 6.0%, 0.6% of sodium bicarbonate and 0.6% of chitin. The predicted value of the swelling performance was 5085.25%, while the actual measured swelling performance was 5358.00%. Additionally, the predicted value of the compression performance was 41.85 kPa, whereas the actual measured real value was 44.45 kPa. The error value between the real and predicted values of the swelling ratio and compression performance was 4.9% and 5.8%, respectively. The relative errors between the predicted and experimental values of the swelling properties and compression performance in the response surface optimization results were between 0–6%, indicating the good reliability of the model. The swelling kinetics conformed to the pseudo second-order kinetic model with R^2^ = 0.997 and the dendritic structure was generated in the hydrogel due to the addition of sodium bicarbonate. FTIR revealed the interaction between individual raw materials in hydrogels. The present study can provide a new method of developing efficient starch-based chlorine dioxide hydrogels for the sustained release of chlorine dioxide.

## 4. Materials and Methods

Cassava starch (Food grade, amylose content 20%, amylopectin content 80%) is from Nanjing GanZhiyuan Sugar Co., Ltd. (Nanjing, China); Xanthan gum (US Pharmacopoeia grade), Polyvinyl alcohol (Mw~205,000), Carboxymethyl cellulose (2500–4500 MPa.s) are all from Shanghai Aladdin Biochemical Technology Co., Ltd. (Shanghai, China); 2,3-epoxypropyltrimethylammonium chloride (Analytical grade) and Chitin (Practical grade) are from Shanghai McLean Biochemical Technology Co., Ltd. (Shanghai, China); Epichlorohydrin (Analytical grade) from Chengdu Kelong Chemical Co., Ltd. (Chengdu, China); Sodium hydroxide (Analytical grade) from Tianjin Damao Chemical Reagent Factory. (Tianjin, China); Absolute ethanol (Analytical grade) from Tianjin Zhiyuan Chemical Reagent Co., Ltd. (Tianjin, China); Sodium bicarbonate (Analytical pure) from Tianjin Kemeiou Chemical Reagent Co., Ltd. (Tianjin, China).

**Figure 9:**
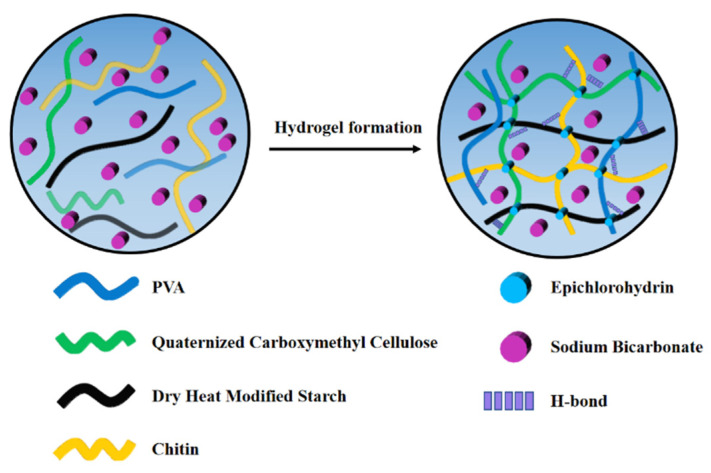
A schematic diagram of the hydrogel formation. Single-factor test: single-factor experiment is used to determine the optimal concentration of dry heat modified starch, quaternized carboxymethyl cellulose, chitin, PVA, epichlorohydrin, sodium bicarbonate. In this test, six concentrations (*w*/*v* % or *v/v* %) of dry heat modified starch (2.4%, 2.6%, 2.8%, 3.0%, 3.2%), quaternized carboxymethyl cellulose (0.48%, 0.64%, 0.8%, 0.96%, 1.12%), chitin (0.4%, 0.6%, 0.8%, 1.0%, 1.2%), PVA (0.6%, 0.8%, 1.0%, 1.2%, 1.4%), epichlorohydrin (3.6%, 4.8%, 6%, 7.2%, 8.4%) and sodium bicarbonate (0.4%, 0.6%, 0.8%, 1.0%, 1.2%) were selected to perform single-factor test. The swelling properties and mechanical properties were measured to determine the effects of each factor on the hydrogel properties, and the factors among these factors that had more significant effects on the swelling properties and mechanical properties of the hydrogel were selected for Box—Behnken design optimization tests.

Preparation of starch-based acid- and alkali-resistant hydrogels: the dry heat-modified starch and quaternized carboxymethyl cellulose were prepared according to previous studies [41,42]. 0.4 g of xanthan gum and 39.6 g of cassava starch were stirred and evenly mixed. The mixture was then placed in a Petri dish, dried at 45 °C for 12 h, and then placed in an oven. After drying at 130 °C for 3 h, the dry heat-modified starch was obtained. Next, a certain amount (2 g) of carboxymethyl cellulose was weighed and fully dissolved in distilled water. Subsequently, 0.5 g of 2,3-epoxypropyltrimethylammonium chloride was added, and the mixture was placed at 60 °C. The reaction was conducted in an oil bath for 24 h; the generated products were washed with absolute ethanol and centrifuged (6000 rpm, 10 °C, 10 min) repeatedly to obtain the quaternized carboxymethyl cellulose. Based on single-factor experiments and Box—Behnken design results. The optimized chitin was added to a solution containing 6.0% (*w*/*v*, V = 25 mL) sodium hydroxide [43]. Next, 1.0% (*w*/*v*) PVA, optimized dry heat-modified starch, quaternized carboxymethyl cellulose, and 0.6% (*w*/*v*) sodium bicarbonate were added and stirred at room temperature (25–27 °C). Subsequently, 6.0% (*v*/*v*) cross-linking agent epichlorohydrin was added and stirred until the oil droplets disappeared. The mixture was then sealed and allowed to stand for 5 h to collect the generated hydrogel. A schematic diagram of gel formation is shown in Figure 9.

Swelling ratio of hydrogels: the dried hydrogel film was weighed (0.25 g, designated as W_d_) and allowed to swell in distilled water (pH = 6.86) for 24 h until the swelling equilibrium was reached. This hydrogel was then weighed (denoted as W_s_); the process was performed for three parallel samples. The hydrogel swelling ratio was then calculated using Equation (7) [40]:

(7) $$\mathrm{SR}=\frac{W_{s}-W_{d}}{W_{d}}\times 100\%$$

where SR is the swelling ratio of the hydrogel (%), W_d_ is the dry hydrogel weight (g), and W_s_ is the wet hydrogel weight (g) after swelling for 24 h.

Mechanical testing: compression tests of the hydrogels were performed using a universal material testing machine (INSTRON-3367). The sample was cut into a cuboid of 18 mm × 18 mm × 15 mm and compressed to the target strain with a 500 N sensor at a speed of 5 mm/min.

Box–Behnken design of swelling properties and compression performance: the Box—Behnken design is a typical design model for the response surface method. This design was utilized in the present study to optimize the parameters of the raw materials that had a substantial impact on the swelling properties and compression performance of the hydrogels: dry heat modified starch, quaternized carboxymethyl cellulose, chitin. The interaction between various parameters was visualized for preparing the hydrogels. As illustrated in Table 5.

Testing and characterization: FTIR spectroscopy was performed in the range of 4000–400 cm^−1^ to determine the functional groups in the starch-based acid- and alkali-resistant hydrogels. The apparent morphology of the starch-based acid- and alkali-resistant hydrogels was determined by SEM. The swelling kinetics of the hydrogel was also determined. The dried hydrogel was accurately weighed to 0.25 g and placed in a buffer solution of pH = 8 at 30 °C. Subsequently, the hydrogel was removed from the liquid, and the surface was dried with filter paper. The hydrogel was then placed on a balance, weighed, and calculated using Equation (8). An average value of the three parallel samples was determined.

(8) $$\mathrm{SR}=\frac{\left(m_{t}-m_{d}\right)}{m_{d}}$$

where m_t_ is the weight of the gel at time t (g) and m_d_ is the weight of the dry gel (g).

## Figures and Tables

**Table 1:** Design and results of the central combination test.

Serial Number	c(Dry Heat-Modified Starch)	c(Quaternized Carboxymethyl Cellulose)	c(Chitin)	Swelling Properties /%	Compression Performance /kPa
1	2.8%	0.8%	0.6%	5032	43.785
2	2.8%	0.96%	0.4%	3560	21.410
3	2.8%	0.8%	0.6%	5094	44.784
4	2.8%	0.64%	0.4%	2690	30.744
5	2.6%	0.8%	0.8%	3170	33.110
6	2.6%	0.96%	0.6%	3509	27.390
7	3%	0.8%	0.8%	2964	19.360
8	2.6%	0.8%	0.4%	2765	23.140
9	2.6%	0.64%	0.6%	1794	37.555
10	3%	0.96%	0.6%	3182	25.230
11	2.8%	0.64%	0.8%	2790	30.175
12	2.8%	0.8%	0.6%	5258	45.980
13	2.8%	0.8%	0.6%	5194	42.445
14	3%	0.64%	0.6%	3055	22.195
15	2.8%	0.96%	0.8%	2895	28.465
16	2.8%	0.8%	0.6%	5134	46.325
17	3%	0.8%	0.4%	3976	23.729

**Table 2:** Analysis of variance (ANOVA) of the test results for the swelling properties of hydrogels.

Source of Variance	Sum of Square	Degree of Freedom	Mean Square	F Value	*p* Value	Salience
Model	18,910,000	9	2,101,000	117.24	<0.0001	**
A-c(Dry heat modified starch)	470,000	1	470,000	26.23	0.0014	**
B-c(Quaternized carboxymethyl cellulose)	991,900	1	991,900	55.35	0.0001	**
C-c(Chitin)	171,700	1	171,700	9.58	0.0174	*
AB	630,400	1	630,400	35.18	0.0006	**
AC	502,000	1	502,000	28.01	0.0011	**
BC	146,300	1	146,300	8.16	0.0244	*
A^2^	4,305,000	1	4,305,000	240.26	<0.0001	**
B^2^	6,539,000	1	6,539,000	364.90	<0.0001	**
C^2^	3,506,000	1	3,506,000	195.62	<0.0001	**
Residual	125,400	7	17,919.85			
Lack of fit	94,811.75	3	31,603.92	4.13	0.1022	Not significant
Pure error	30,627.20	4	7656.80			
Total difference	19,030,000	16				

Note: *p* < 0.05 indicates significant difference *; *p* < 0.01 indicates highly significant difference **.

**Table 3:** ANOVA of the test results for the hydrogel compression performance.

Source of Variance	Sum of Square	Degrees of Freedom	Mean Square	F Value	*p* Value	Salience
Model	1428.82	9	158.76	76.17	<0.0001	**
A-c(Dry heat modified starch)	117.67	1	117.67	56.45	0.0001	**
B-c(Quaternized carboxymethyl cellulose)	41.29	1	41.29	19.81	0.0030	**
C-c(Chitin)	18.26	1	18.26	8.76	0.0211	*
AB	43.56	1	43.56	20.90	0.0026	**
AC	51.40	1	51.40	24.66	0.0016	**
BC	14.53	1	14.53	6.97	0.0334	*
A^2^	397.60	1	397.60	190.77	<0.0001	**
B^2^	197.79	1	197.79	94.90	<0.0001	**
C^2^	430.50	1	430.50	206.55	<0.0001	**
Residual	14.59	7	2.08			
Lack of fit	4.39	3	1.46	0.57	0.6621	Not significant
Pure error	10.20	4	2.55			
Total difference	1443.41	16				

Note: *p* < 0.05 indicates significant difference *; *p* < 0.01 indicates highly significant difference **.

**Table 4:** Model fitting parameters.

Model	Equation	R^2^	k	q
pseudo first-order kinetic model	Q_e_ − exp(ln(Q_e_) − k_1_ × t)	0.989	0.10 ± 0.006	8.09 ± 0.15
pseudo second-order kinetic model	t/((1/(k × Q_e_^2^)) + t/Q_e_)	0.997	0.009 ± 0.0005	10.88 ± 0.18

**Table 5:** Central composite test factor level coding table.

		Range and Levels (Coded)		
Factors	Variable	−1	0	1
c(Dry heat modified starch)	A	2.6%	2.8%	3%
c(Quaternized carboxymethylcellulose)	B	0.64%	0.8%	0.96%
c(Chitin)	C	0.4%	0.6%	0.8%

## Data Availability

Not applicable.
